# Ligand Shaping in Induced Fit Docking of MraY Inhibitors. Polynomial Discriminant and Laplacian Operator as Biological Activity Descriptors

**DOI:** 10.3390/ijms18071377

**Published:** 2017-06-27

**Authors:** Claudiu N. Lungu, Mircea V. Diudea, Mihai V. Putz

**Affiliations:** 1 Department of Chemistry, Faculty of Chemistry and Chemical Engineering, Babes-Bolyai University, 400028 Cluj, Romania; lunguclaudiu5555@gmail.com (C.N.L.); diudea@gmail.com (M.V.D.); 2 Laboratory of Computational and Structural Physical-Chemistry for Nanosciences and QSAR, Department of Biology-Chemistry, West University of Timisoara, Pestalozzi Str. 16, 300115 Timisoara, Romania; 3 Laboratory of Renewable Energies-Photovoltaics, R&D National Institute for Electrochemistry and Condensed Matter, Dr. A. Paunescu Podeanu Str. No. 144, 300569 Timisoara, Romania

**Keywords:** antibiotics, docking, Quantitative Structure Activity Relationship (QSAR), MraY, manifold

## Abstract

Docking—i.e., interaction of a small molecule (ligand) with a proteic structure (receptor)—represents the ground of drug action mechanism of the vast majority of bioactive chemicals. Ligand and receptor accommodate their geometry and energy, within this interaction, in the benefit of receptor–ligand complex. In an induced fit docking, the structure of ligand is most susceptible to changes in topology and energy, comparative to the receptor. These changes can be described by manifold hypersurfaces, in terms of polynomial discriminant and Laplacian operator. Such topological surfaces were represented for each MraY (phospho-MurNAc-pentapeptide translocase) inhibitor, studied before and after docking with MraY. Binding affinities of all ligands were calculated by this procedure. For each ligand, Laplacian and polynomial discriminant were correlated with the ligand minimum inhibitory concentration (MIC) retrieved from literature. It was observed that MIC is correlated with Laplacian and polynomial discriminant.

## 1. Introduction

Phospho-MurNAC-pentapetide translocase (MraY) is an enzyme involved in bacterial cell wall biosynthesis. Its major role is the transfer of peptidoclycan precursor phospho-MurNAc pentapetide to undecaprenyl phosphate [1]. Mray is the target for five families of nucleosides which are natural antibacterials: the tunicamycins, liposydomycins, muraymycins, and capuraymicins [2]. These compounds act as inhibitors of MraY. This enzyme is regarded as an ideal target for novel antibiotics due to its crucial role in generating the cellular envelope and because it lacks in mammalian cells. Different classes of inhibitors have been studied. None of these has entered into clinical trials due to difficulties in delivering the compounds across the membrane [3]. Thus, assessing the bioactivity of novel candidates for MraY inhibition is important. In this study, a computational method of determining the bioactivity of five MraY inhibitors is presented. This method is based on descriptors that relate to molecular recognition process. Molecular recognition is simulated using molecular docking. In physiological conditions, when the interaction between a ligand and a receptor occurs, ligand and receptor “model” to each other their structure in order to form a complex able to act on a living tissue. Because the goal of this study is to develop a methodology to predict the bioactivity based on the ligand structure, the receptor (MarY) was considered rigid in simulating the molecular recognition process. The prediction is based on the docked ligand configuration.

In describing a chemical structure, molecular descriptors are used. A common issue of molecular descriptor is their lack of “sensitivity” when describing the dynamic of a certain compound. The majority of descriptors show the same values for the free and docked conformation of a certain compound (i.e., show degenerate values—see Table 1). Another issue of molecular descriptors is the characterization of a whole process by a single numerical value. To solve this matter, a molecular topological surface was used. The surface was generated using the Cartesian coordinates of the atoms of ligands in the free (undocked) and docked form. The topological surface of a compound is a shape according to its specific interaction with the receptor and eventually correlates with the biological effect. Surface obtained was characterized using the polynomial discriminant [4] and Laplacian operator [5]. The two “operations” describe different properties of the topological surface. Polynomial discriminant, intimately related to the roots of the chosen second-degree polynomial, has a geometric descriptive value correlated with the shape of the manifold [6]. The Laplacian operator is related to the gradient of the surface [7,8], used to describe Riemannian manifolds [9].

The surface as such obtained represents a special type of surface namely a two-dimensional manifold. In mathematics, a manifold is a topological space that locally resembles Euclidean space near each point. Each point of an *n*-dimensional manifold has a neighborhood that is homeomorphic to the Euclidean space (there is a continuous function between the two spaces that has a continuous inverse function) of dimension *n*. Thus, a manifold has the property of being locally Euclidean, property preserved by a local homeomorphism [10]. On such properties, minimization docking algorithms are based [11]. A polynomial discriminant is the product of the square of the differences of the polynomial roots. In algebra, the discriminant of a polynomial is a polynomial function of its coefficients. The discriminant is widely used in number theory through its generalization as the discriminant of a number field [12]. The discriminant of a polynomial is identified only up to a constant factor and several different normalizations can be used [13]. For a quadratic equation a_2_z^2^ + a_1_z + a_0_ = 0 the discriminant of a univariate polynomial *p*(*x*) is given by the expression D_2_ = (a_1_)^2^ − 4a_0_a_2_. The typical use of discriminants in algebraic geometry is the study of algebraic curves and more generally algebraic hypersurfaces. If *V* is such a curve or hypersurface, *V* is defined as the zero set of a multivariate polynomial. This polynomial may be considered a univariate polynomial in one of the indeterminates, with polynomials in the other indeterminates’ coefficients. The discriminant with respect to the selected indeterminate defines a hypersurface *W* in the space of other indeterminates. The points of *W* are exactly the projection of the points of V which either are singular or have a tangent hyperplane that is parallel to the axis of the selected indeterminate. If *f* is a bivariate polynomial in X and Y with real coefficients, such that *f* = 0, is the implicit equation of a plane algebraic curve. Computation of the roots of *Y*-discriminant and *X*-discriminant allows one to compute all of the remarkable points of the curve, except the inflection points. Laplacian (i.e., Laplace operator) is a differential operator given by the divergence of the gradient of a function on Euclidean space. The Laplacian Δ*f*(*p*) of a function *f* at a point *p*, up to a constant depending on the dimension, is the rate at which the average value of *f* over spheres centered at *p* deviates from *f*(*p*) as the radius of the sphere grows. In the Cartesian coordinate system, the Laplacian is given by the sum of second partial derivatives of the function *f* with respect to each independent variable. Laplacian represents the flux density of the gradient flow of a function. For instance, the net rate at which a chemical dissolved in a fluid moves toward or away from some point is proportional to the Laplacian of the chemical concentration at that point; expressed symbolically, the resulting equation is the diffusion equation. For these reasons, it is extensively used in sciences for modeling physical phenomena like energy minimization, where the solutions to Δ*f* = 0 in a region U are functions that make the Dirichlet energy functional stationary.

## 2. Results

**Figure 1:**
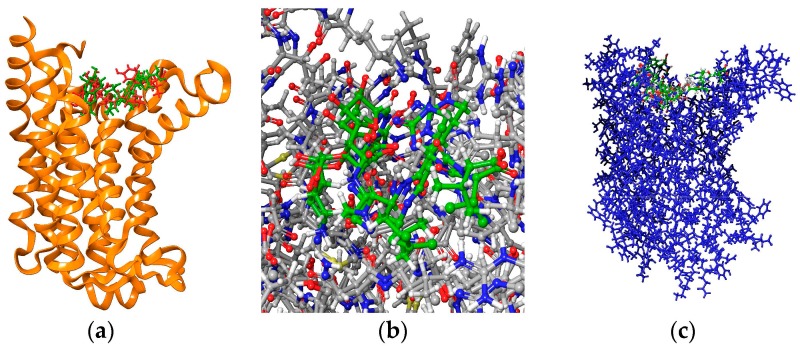
5ckr MraY in complex with Muraymicin D2. (**a**) Muraymycin D2 redocked and superposed on the same binding site; a general view MraY shown as ribbons colored in alloy orange, crystallographic pose-green, redocked pose-red; (**b**) superposition of the two molecules of Muraymycin, colored in green; (**c**) all five ligands docked at the MraY binding site.

Docking procedure was performed successfully. The receptor (MraY) was considered rigid and the ligands were considered mobile. For each ligand, the best conformational poses were recorded for their binding affinities. All ligands were docked at the designated binding site. In Figure 1c, best poses for the studied compounds are shown in green color at the MraY binding pocket. It can be observed that no compound is docked at another site or is left out. For testing the ability of docking to predict the correct (i.e., bioactive) conformation re-docking of Muraymycin D on MraY proved to be successful. As shown in Figure 1a,b, the two molecules of Muraymycin D2 (one measured crystallographic and one predicted by docking) superposed almost perfectly.

Computed descriptors and their values are shown in Table 1. 

Table 1 shows well-known molecular descriptors: principal moment of inertia (PM) chosen for its ability of sensing ligand shape changes, Wiener index (W) chosen for its relation to molecular branching and van der Waals surface area, and the molecular topological index (MTI) a vertex valence-weighted analogue of Wiener index. Both W and MTI are based on distance matrix (W) and adjacency and distance matrix (MT) thus they do not “feel” changes in atom positions (x, y, z coordinates) consequently do not discriminate between the docked and undocked (free) state. PM shows distinct values for free and docked form while it is based on the distance of the *i*th atomic nucleus from the *k*th main rotational axes (k = x, y or z). These descriptors are given here to illustrate the difficulty of finding proper descriptors to distinctly characterize the both states (undocked and docked, respectively) and to demonstrate the reliability of a descriptor based on all or one of x, y, z coordinates in describing such states. Laplacian and polynomial discriminant based on topological surfaces (generated using x, y, z, Cartesian coordinates) are such descriptors.

**Figure 2:**
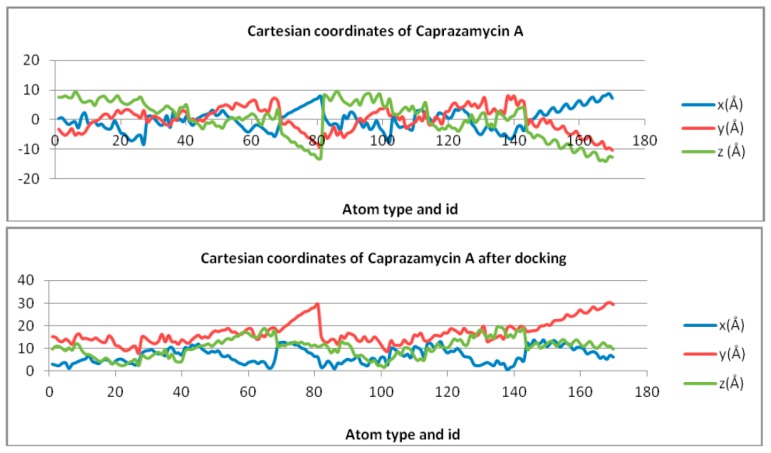
Cartesian coordinates of Caprazamycin A, undocked (top) and docked (bottom), represented as scatter plots.

**Figure 3:**
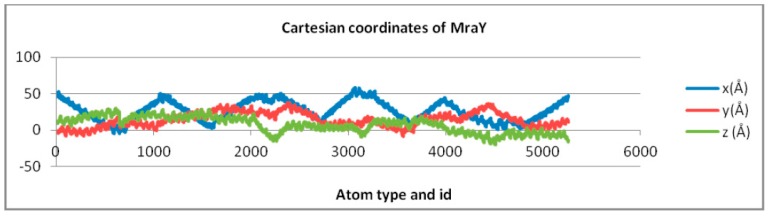
Cartesian coordinates of MraY represented as a scatter plot.

Cartesian coordinates for the undocked and docked Caprazamycin A and MraY receptor are given in Table 2 and represented in Figure 2 and Figure 3 (see supplemental materials for the rest of graphs).

**Figure 4:**
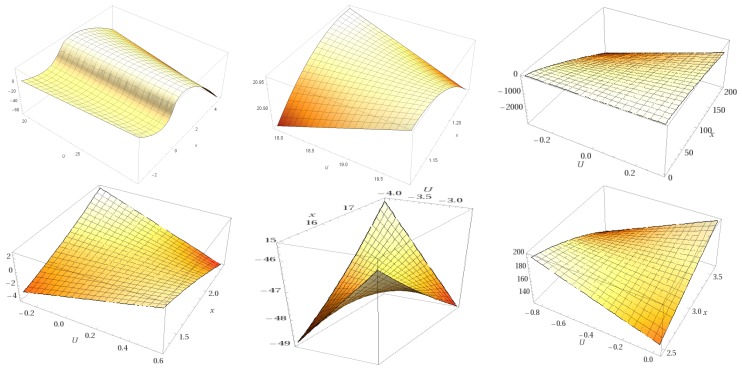
Topological manifolds of free molecules showing (from left to right, first and second row): Caprazamycin A, Liposidomycin B, MuraymycinCl, Mureidomycin A, Tunicamycin I, and MraY respectively.

Figure 4 illustrates the topological surfaces for all the structures (five ligands and one receptor) in their free state energetically minimized, represented as manifolds [14].

**Figure 5:**
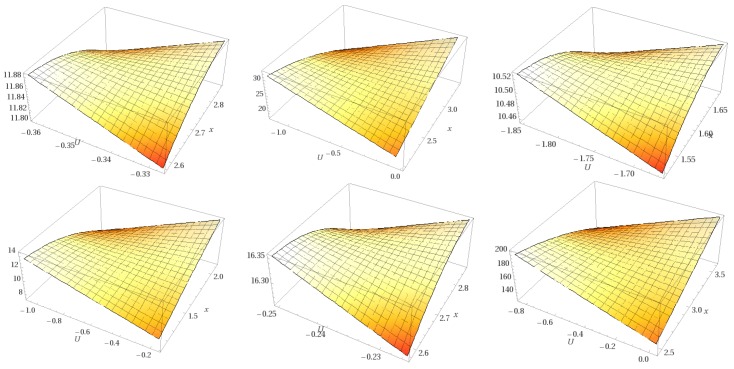
Topological manifolds, after docking (from left to right, first and second row): Caprazamycin A, Liposidomycin B, MuraymycinCl, Mureidomycin A, Tunicamycin I, and MraY, respectively.

Manifolds resulted after a rigid docking with MraY, together with MraY itself, are represented in Figure 5.

Equation resulted by characterizing coordinates trend lines are represented in Table 3 and Table 4 together with the Laplacian operator and polynomial discriminant calculated for second and fourth degree polynomial equation, respectively. 

**Figure 6:**
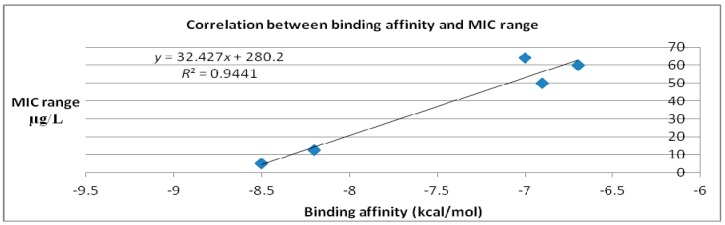
Plot of binding affinity calculated computationally vs. MIC literature data (after docking case, Table 2).

**Figure 7:**
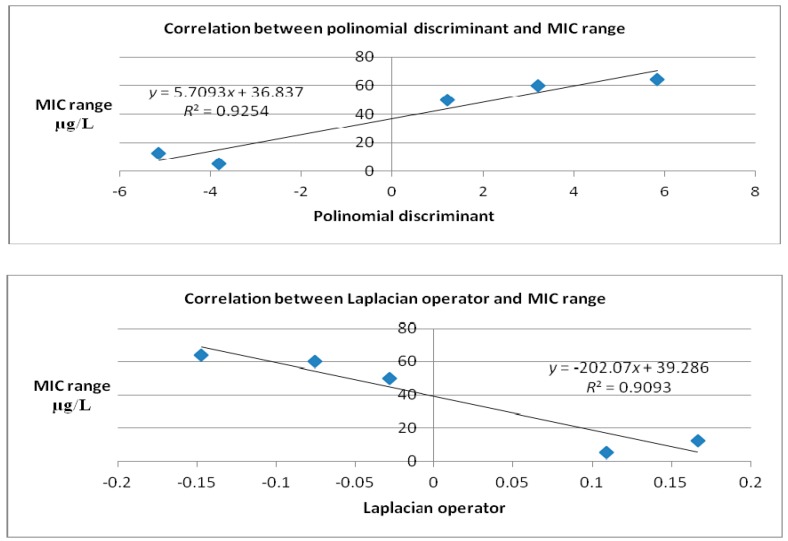
Correlation between polynomial discriminant (top) and Laplacian (bottom) for the second degree (after docking case, Table 2) and MIC values of: Caprazamycin A, Liposidomycin B, MuraymycinCl, Mureidomycin A, Tunicamycin I.

Correlation between binding affinity calculated computationally and MIC values retrieved from literature (Table 5) is represented in Figure 6, while the correlation between MIC and Laplacian operator and polynomial discriminant are shown in Figure 7.

## 3. Discussion

**Figure 8:**
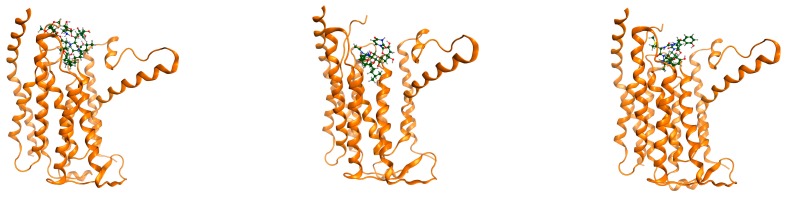

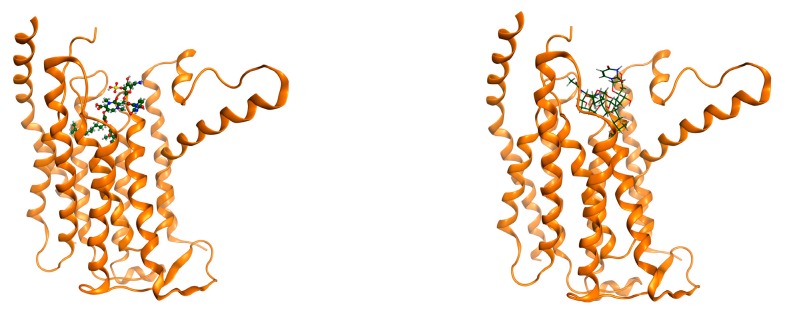
MraY in complex with (from left to right, top and bottom rows): Caprazamycin A, Liposidomycin B, Muraymycin Cl, Mureidomycin A, Tunicamycin I. MraY shown as ribbons colored in alloy orange. Muraymycin D displayed as cylindrical bonds C—green, H—grey, O—red, N—blue.

Cartesian coordinates are different in the minimized energy free state compared to the docked state, when receptor–ligand complexes are formed (Figure 8).

Topology of hypersurfaces also differs; after docking, the topological space changes significantly. The topological space of the five docked ligands tends to “occupy” approximately the same coordinates. This means that ligands in a rigid body docking are shaped according to the binding site geometry. Surfaces obtained by the methodology discussed above and represented in Figure 6, show a high degree of similarity. There exists an energetically optimal conformation for the topological manifold of each ligand. The topological spaces of ligands, in the undocked (i.e., free, energetically minimized ligand) and docked (in a complex with MraY) state, respectively, are Euclidean-Hausdorff spaces, described as topological manifolds. Induced fit docking Cartesian coordinates and the subsequent topological spaces are modeled by the intra- and intermolecular forces, according to the type of interaction: (i) electrostatic forces (due to the charges residing in the matter); (ii) electrodynamics forces; (iii) most widely occurring van der Waals interactions; (iv) steric forces (originating in entropy, manifested, e.g., in solvation processes, particularly in water molecules displacement out of the binding site); (v) hydrogen bonds; (vi) hydrophobic interactions, etc. 

Translation and rotation of a molecule relative to another one involves six degree of freedom and there are, in addition, conformational degrees of freedom of both the ligand and protein; the solvent may also play a significant role in determining the protein–ligand geometry, often ignored in this kind of computations. Topological surfaces represented here are those of the most favorable molecular conformations. The topological surface of MraY alone remains constant; having the receptor atoms fixed, there must be a conformation translated into a topological surface or a 2-type manifold that is the most favorable for the biological action.

Binding affinity, expressed in kcal/mol, is a global thermodynamic parameter for evaluating the docking process [20]. Both 2-type manifold and binding affinity are “modeled” by the same forces, that is why the correlation between the polynomial discriminant and Laplacian operator (describing the manifold) and the binding affinity, on the other part, is significant. For any biological process that implies, as the mechanism of action, formation of a ligand–receptor complex, the variation of free energy, ΔG taken as a global measure of the docking process, is highly correlated with the biological activity. In the case of MarY inhibitors herein studied, the biological activity can be expressed by minimal inhibitory concentration (MIC). The range of MIC is the maximal distance where an antibiotic shows a bacterial growth inhibition.

## 4. Material and Methods

**Figure 9:**
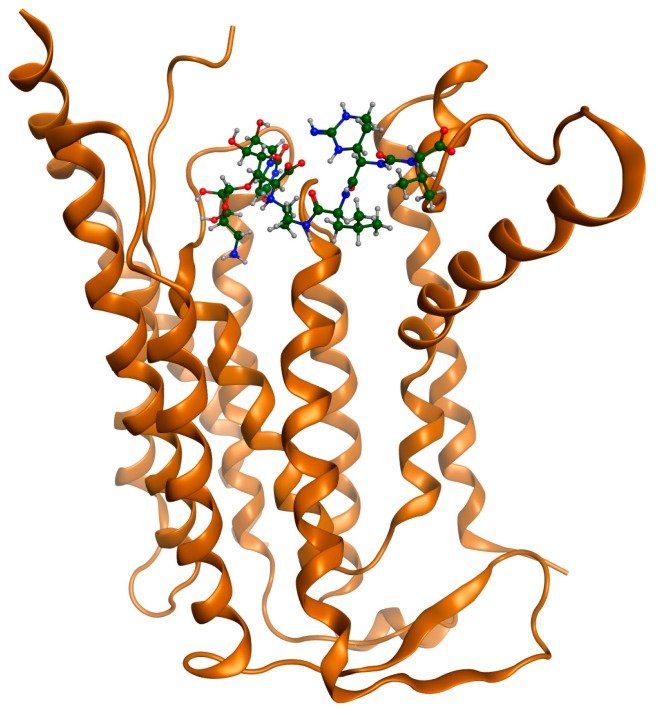
5ckr showing MraY in complex with Muraymycin D2. MraY shown as ribbons colored in alloy orange. Muraymycin D displayed as cylindrical bonds C—green, H—grey, O—red, N—blue.

Structures of five representative class members of MraY inhibitors were studied: Caprazamicyn A, Liposidomicyn B, Muraymycin Cl, Mureidomycin A, Tunicamycin I. All structures were retrieved from [21]. Structures can alternatively be retrieved as follows: Caprazamicyn A retrieved from [22], Liposidomicyn B from Pub Chem CID 443576, Muraymycin Cl from [23], Mureidomycin A by Chemical Entities of Biological Interest, ChEBI ID: 29632, Tunicamycin I by Pub Chem CID 56927848. The structures were built by generating the 3D structures from the 2D representations retrieved using the sources listed above. This methodology was used in order to have a uniform construction method. LigPrep software integrated in Schrodinger 2009 package was used to prepare the ligands. Molecules were energetically minimized, in vacuum, using Ligprep (Schrodinger suite, New York, NY, USA). Molecular force field used was OPLS2005 (Schrodinger suite, New York, NY, USA). MraY molecule was chosen as a receptor. The structure was retrieved using PDB 5ckr, which represents MraY in complex with Muraymycin D2. Ligand and water molecules were removed from the model. The model was further prepared using Protein Preparation Wizard as follows: bond orders were assigned, hydrogen atoms were added, amino acid loops were corrected using Prime (a protonated state at pH 7.4 was thus generated). The structure was energetically minimized using OPLS2005 force field, in vacuum. Complexes between MraY used as a receptor and the discussed ligands were generated using a docking procedure. Software used was AutoDockVina [24]. Receptor (MraY) was considered fixed, ligands were considered mobile. The active site of MraY was retrieved from the literature [25,26] and using the PDB structure 5ckr [27] (see Figure 9). Binding affinities were recorded and expressed in kcal/mol. The docking procedure is as follows. PDB file containing MraY without the ligand was opened. Ligands, initially in SDF format were converted in PDB, by PyMol. As Autodock Vina requires, pdbqt files were prepared from the pdb files of protein (MraY) and ligands. Grid box, enclosing all the selected residues, had the following coordinates: center_x = 9.74; center_y = 16.09; center_z = 4.86, with a spacing of 0.375. Size of the rectangular box was set to: size_x = 25, size_y = 25, size_z = 25. All data are expressed in Å. After docking procedure was running, the best pose was considered for each ligand, in respect of collecting the binding affinity values (kcal/mol). When the binding pocket is unknown, mapping of the receptor is made in respect of finding several most active sited, then decide which one is appropriate for a particular case (ligand and receptor, respectively). However, AutoDock or related software possess tools for detecting cavities and binding pockets. For example, in AutoDock4.2 (The Scripps research Institute & Olso Laboratory USA; San Diego, CA, USA), one can build a grid volume big enough to cover the entire surface of the protein, using a larger grid spacing than the default value of 0.375 Å, and more grid points in each dimension. Then preliminary docking experiments can be performed with AutoDock4.2 (The Scripps research Institute & Olso Laboratory USA; San Diego, CA, USA) to see if there are particular regions of the protein that are preferred by the ligand. This is sometimes referred to as ‘blind docking’. Then, in a second round of docking experiments, smaller grids can be built around these potential binding sites and dock in them smaller grids. If the protein is very large, then the protein can be break up (divided) into overlapping grids and dock into each of these grid sets, e.g., one covering the top region, one covering the bottom region, and one covering the middle [28]. After identifying the binding site, the docking is carried on AutoDock4.2 (The Scripps research Institute & Olso Laboratory USA; San Diego, CA, USA) or AutoDock Vina as described above.

Docking accuracy in retrieving biologically significant poses was checked by docking all the ligands at the same binding site and by docking Muraymycin D2 lingand with the 5ckr PDB crystallography model in order to compare position of Muraymycin D2 docked with its crystallographic determined coordinates.

However, docking results are the outcome of a series of “in silico computations”. Results differ irrespective of docking software, search algorithm, or scoring functions. These differences are due to semiempirical calculations, randomized algorithms, and molecular mechanics approach which are implemented in all docking algorithms used. A series of comparative studies between docking software have shown major differences in respect to pose matching and errors. In such a study (Ramierez and Caballero 2016) it was demonstrated a major difference between AutoDock and Glide software in predicting binding affinities for a series of enantiomer pairs which were previously experimentally determined. The results of this study, strongly based on the accuracy of docking, should be considered in respect with this statements [29].

Common descriptors show degeneracy in treating the undocked and docked state of a ligand series. Such descriptors—like principal moment of inertia, PM [30]; Wiener index, W [31]; molecular topological index; and MTI [32]—were computed and listed in Table 1.

Topological coordinates for all ligands and their pharmacological target MraY were represented using scatter plots. The trend for each coordinate was expressed as a trendline logarithmic equation. Reunion of logarithmic equations was represented as an integrated log(x) function. The procedure was applied to all molecules under study. For example, for Caprazamycin A, the logarithmic equation generating the surface is of the form: Y = ∫(0.9741ln(x) − 4.0486U × 0. 1524ln(x) − 0.6333U − 3.588ln(x) + 14.913)dx. Using the above equation, 3D plots were generated for all the five structures.

Cartesian coordinates were represented in a unique 3D plot, in order to compute a polynomial equation able to characterize all the three coordinates at a time. Using a second degree polynomial function, the Laplacian operator and polynomial discriminant were tailed for both the undocked and docked ligand structures. These operations were performed for the receptor molecule as well. Resulting data were thus compared with binding affinities and minimum inhibitory concentration MIC (µg/mL) against acid fast bacteria [33].

## 5. Conclusions

Ligand topological surface, after docking, is clearly modeled by the receptor. Topological surface of the ligand is a two-dimensional manifold, modeled by intra- and intermolecular forces involved in docking. Laplacian operator and polynomial discriminant of the second degree equation (x, y, z-trends) can be used to distinctly characterize the topological surface of each ligand. Cartesian coordinate equations are in strong correlation with the binding affinity (computed in silico) and MIC range. These operators, calculated for a 2-degree topological manifold of docked ligands can be used in predicting bioactivity on a path involving the ligand–receptor complex.

## Figures and Tables

**Table 1:** Topological descriptors computed for MraY free (undocked) and docked ligands (see text for details).

State	Undocked			Docked		
Ligand	PM	W	MTI	PM	W	MTI
Caprazamycin A	10,307.536	34,977	236,428	17,708.229	34,977	236,428
Liposidomycin B	8447.889	23,994	161,237	10,855.062	23,994	161,237
Muraymycin Cl	8196.615	21,822	143,843	9364.920	21,822	143,843
Mureidomycin A	7980.791	16,639	113,829	9722.197	16,639	113,829
Tunicamycin I	5628.636	14,696	99,741	11,318.951	14,696	99,741

**Table 2:** Logarithmic equations for free and docked ligands.

**Free Molecule**	***X* Coordinate**	***Z* Coordinate**	***Y* Coordinate**
Caprazamycin A	Y = 0.9741ln(x) − 4.0486	Y = 0.1524ln(x) − 0.6333	Y = −3.588ln(x) + 14.913
Liposidomycin B	Y = 0.2486ln(x) − 4.9995	Y = −2.49ln(x) + 8.0632	y = −2.371ln(x) + 6.8077
Muraymycin Cl	Y = 0.3637ln(x) + 8.2632	Y = − 2.35ln(x) + 9.3795	Y = −0.105ln(x) + 0.4055
Mureidomycin A	Y = 2.3747ln(x) − 7.7925	Y = 1.7282ln(x) − 5.4429	Y = −0.13ln(x) + 0.4852
Tunicamycin I	Y = −1.382ln(x) − 1.382	Y = 1.219ln(x) + 3.022	Y = −1.325ln(x) + 1.95
MraY	Y = −0.991ln(x) + 34.353	Y = 3.639ln(x) − 12.969	Y = −6.486ln(x) + 56.661
**Docked Molecule**	***X* Coordinate**	***Z* Coordinate**	***Y* Coordinate**
Caprazamycin A	Y = 1.1474ln(x) + 2.1222	Y = 2.2825ln(x) + 7.4555	y = 1.4935ln(x) + 4.3625
Liposidomycin B	Y = 0.6125ln(x) + 8.986	Y = 1.8278ln(x) + 9.8712	Y = 0.1306ln(x) + 9.6344
Muraymycin Cl	Y = 1.9153ln(x) + 2.4093	Y = −0.492ln(x) + 22.764	Y = 0.1996ln(x) + 7.3225
Mureidomycin A	Y = 0.9779ln(x) + 6.4303	Y = −0.93ln(x) + 19.552	Y = 0.6179ln(x) + 7.1176
Tunicamycin I	Y = 0.6101ln(x) + 10.26	Y = 1.5282ln(x) + 14.268	Y = 0.851ln(x) + 6.0062
MraY	Y = −0.991ln(x) + 34.353	Y = 3.639ln(x) − 12.969	Y = −6.486ln(x) + 56.661

**Table 3:** Polynomial second degree equations used to calculate the Laplacian operator and polynomial discriminant.

**Molecule**	**Cartesian Equation before Docking**	**Cartesian Equation after Docking**
Caprazamycyn A	Y = −0.0453x^2^ − 0.7281x + 1.5735	Y = −0.0143x^2^ + 0.670x + 13.167
Liposidomycin B	Y = 0.1597x^2^ + 0.9449x − 1.1911	Y = −0.0736x^2^ + 2.5211x − 1.7814
Muraymycin Cl	Y = 0.0151x^2^ + 0.4133x + 4.8176	Y = −0.0378x^2^ + 0.6574x + 18.339
Mureidomycin A	Y = 0.0179x^2^ + 0.6583x − 0.0096	Y = 0.0833x^2^ − 1.6429x + 23.528
Tunicamycin I	Y = −0.0871x^2^ + 0.7366x + 2.0081	Y = 0.0546x^2^ − 0.6779x + 19.579
MraY	Y = 0.0003x^2^ − 0.0085x + 14.496	Y = 0.0003x^2^ − 0.0085x + 14.496
**Polynomial Discriminant**		
Molecule	Cartesian equation before docking	Cartesian equation after docking
Caprazamycin A	0.815248	1.20205
Liposidomicyn B	1.65371	5.8315
Muraymycin Cl	−0.120166	3.20503
Mureidomycin A	0.434046	−5.14041
Tunicamycin I	1.2422	−3.81651
MraY	−0.017323	−0.017323
**Laplacian**		
Molecule	Cartesian equation before docking	Cartesian equation after docking
Caprazamycin A	−0.0906	−0.0286
Liposidomicyn B	0.3194	−0.1472
Muraymycin Cl	0.0302	−0.0756
Mureidomycin A	0.0358	0.1666
Tunicamycin I	−0.1742	0.1092
MraY	0.0006	0.0006

**Table 4:** Polynomial fourth degree equations used to calculate and the polynomial discriminant.

**Molecule**	**Cartesian Equation before Docking**	**Cartesian Equation after Docking**
Caprazamycin A	Y = −0.001x^4^ − 0.0074x + 0.0146x^2^ − 0.4585x + 1.3125	Y = 0.0052x^4^ − 0.1596x^3^ + 1.6554x^2^ − 6.0837x + 21.636
Liposidomycin B	Y = −0.0163x^4^ − 0.3471x^3^ − 2.3041x^2^ − 5.5628x − 6.0698	Y = −0.0107x^4^ + 0.3649x^3^ − 4.2848x^2^ + 20.373x − 18.692
Muraymycin Cl	Y = 0.0008x^4^ − 0.0256x^3^ + 0.1962x^2^ + 0.4938x + 3.5622	Y = 0.0038x^4^ − 0.1135x^3^ + 1.017x^2^ − 2.1836x + 16.887
Mureidomycin A	Y = −0.0014x^4^ − 0.0139x^3^ + 0.1119x^2^ + 1.1734x − 0.854	Y = −0.0039x^4^ + 0.1384x^3^ − 1.6016x^2^ + 6.5958x + 10.301
Tunicamycin I	Y = 0.0013x^4^ − 0.0044x^3^ − 0.1304x^2^ + 0.8329x + 2.1574	Y = 0.0052x^4^ − 0.2714x^3^ + 5.2418x^2^ − 43.377x + 146.8
MraY	Y = −0.0001x^4^ + 0.0032x^3^ − 0.1071x^2^ + 1.1968x + 11.556	Y = −0.001x^4^ + 0.0032x^3^ − 0.1071x^2^ + 1.1968x + 11.556
**Polynomial Discriminant**		
Molecule	Cartesian equation before docking	Cartesian equation after docking
Caprazamycin A	−2.80872 × 10^−6^	0.181815
Liposidomycin B	−0.0183835	−1.11938
Muraymycin Cl	0.0000417636	0.0936006
Mureidomycin A	−0.0000169638	−0.0829902
Tunicamycin I	−0.0000339967	0.3223
MraY	−2.97825 × 10^−6^	−2.97825 × 10^−6^

**Table 5:** Binding affinity towards MraY docking and minimum inhibitory concentration (MIC) literature values for the studied ligands.

Molecules	Binding Affinity (kcal/mol)	MIC Range (μg/mL)
Caprazamycin A	−6.9	50 [15,16]
Liposidomycin B	−7	64 [17]
Muraymycin Cl	−6.7	60 [18]
Mureidomycin A	−8.2	12.5 [19]
Tunicamycin I	−8.5	5 [19]

## Supplementary Materials

Supplementary materials can be found at www.mdpi.com/1422-0067/18/7/1377/s1.
