# Supplementary material for: Ligand Shaping in Induced Fit Docking of MraY Inhibitors. Polynomial Discriminant and Laplacian Operator as Biological Activity Descriptors
Source: Int J Mol Sci. 2017 Jun 27;18(7):1377. doi: 10.3390/ijms18071377 (PMC5535870; doi:10.3390/ijms18071377)
Supplement: Supplementary file 1 [file ijms-18-01377-s001.zip › ijms-192270 supplementary.doc]

**Supplementary Material: Ligand shaping in induced fit type docking of MraY inhibitors. Polynomial discriminant and Laplacian operator as biological activity descriptor**

Claudiu N. Lungu, Mircea V. Diudea, Mihai V. Putz

Cartesian coordinates of all molecular structures.
